# A soybean quantitative trait locus that promotes flowering under long days is identified as *FT5a*, a *FLOWERING LOCUS T* ortholog

**DOI:** 10.1093/jxb/erw283

**Published:** 2016-07-15

**Authors:** Ryoma Takeshima, Takafumi Hayashi, Jianghui Zhu, Chen Zhao, Meilan Xu, Naoya Yamaguchi, Takashi Sayama, Masao Ishimoto, Lingping Kong, Xinyi Shi, Baohui Liu, Zhixi Tian, Tetsuya Yamada, Fanjiang Kong, Jun Abe

**Affiliations:** ^1^Research Faculty of Agriculture, Hokkaido University, Sapporo, Hokkaido 060-8589, Japan; ^2^The Key Laboratory of Soybean Molecular Design Breeding, Northeast Institute of Geography and Agroecology, Chinese Academy of Sciences, Harbin 150081, China; ^3^Hokkaido Research Organization Tokachi Agricultural Experiment Station, Memuro, Hokkaido 082-0081, Japan; ^4^National Institute of Agrobiological Sciences, Kannondai, Ibaraki 305-8602, Japan; ^5^State Key Laboratory of Plant Cell and Chromosome Engineering, Institute of Genetics and Developmental Biology, Chinese Academy of Sciences, Beijing 1001014, China

**Keywords:** *FLOWERING LOCUS T*, flowering time, near-isogenic line, photoperiod sensitivity, quantitative trait locus, SNP calling, soybean.

## Abstract

*FLOWERING LOCUS T* (*FT*) is an important floral integrator whose functions are conserved across plant species. In soybean, two orthologs, *FT2a* and *FT5a*, play a major role in initiating flowering. Their expression in response to different photoperiods is controlled by allelic combinations at the maturity loci *E1* to *E4*, generating variation in flowering time among cultivars. We determined the molecular basis of a quantitative trait locus (QTL) for flowering time in linkage group J (Chromosome 16). Fine-mapping delimited the QTL to a genomic region of 107kb that harbors *FT5a*. We detected 15 DNA polymorphisms between parents with the early-flowering (*ef*) and late-flowering (*lf*) alleles in the promoter region, an intron, and the 3′ untranslated region of *FT5a*, although the *FT5a* coding regions were identical. Transcript abundance of *FT5a* was higher in near-isogenic lines for *ef* than in those for *lf*, suggesting that different transcriptional activities or mRNA stability caused the flowering time difference. Single-nucleotide polymorphism (SNP) calling from re-sequencing data for 439 cultivated and wild soybean accessions indicated that *ef* is a rare haplotype that is distinct from common haplotypes including *lf*. The *ef* allele at *FT5a* may play an adaptive role at latitudes where early flowering is desirable.

## Highlight

We identified different expression levels of *FT5a*, an ortholog of *FLOWERING LOCUS T*, as the molecular basis of a quantitative trait locus that promotes flowering under long-day conditions in soybean.

## Introduction

Time to flowering and maturation influences the productivity, adaptability, and quality of seed crops. Flowering time is determined by the integration of signals from external stimuli (such as photoperiod and temperature) and internal conditions (such as plant age and the amount of gibberellic acid), which converge on the regulation of *FLOWERING LOCUS T* (*FT*), a long-sought systemic floral inducer (Song *et al.*, 2013). Responses of flowering to photoperiod may be one of the major determinants of adaptation to different daylengths, in particular for plants in temperate zones.

Soybean [*Glycine max* (L.) Merr.], a facultative short-day (SD) plant, is cultivated in a broad range of latitudes, although each cultivar is grown in a very narrow latitude range (Watanabe *et al.*, 2012). This wide adaptability to growing seasons and regions is generated by genetic diversity in flowering responses to various external and internal signals. Ten major genes, *E1* to *E9* and *J*, and a number of quantitative trait loci (QTLs) have been reported to be involved in the control of flowering in soybean. The molecular bases of *E1*–*E4* and *E9* are known, and their functions in the photoperiod responses of flowering have been characterized (Liu *et al.*, 2008; Watanabe *et al.*, 2009, 2011; Xia *et al.*, 2012; Xu *et al.*, 2015; Zhai *et al.*, 2015; Zhao *et al.*, 2016). *E1* encodes a putative transcription factor with a bipartite nuclear localization signal and a region distantly related to the B3 DNA-binding domain; E1 suppresses the expression of the soybean *FT* orthologs *FT2a* and *FT5a* (Xia *et al.*, 2012). Night-break (NB) experiments and experiments with transitions between light and dark phases have revealed that the induction of *E1* expression requires light given at the right time in the circadian rhythm (Xu *et al.*, 2015). This light-regulated *E1* expression is mediated by two phytochrome A (PHYA) proteins, E3 (GmPHYA3) and E4 (GmPHYA2) (Liu *et al.*, 2008; Watanabe *et al.*, 2009; Xia *et al.*, 2012). In plants with the double-recessive *e3*/*e4* genotype, *E1* expression is not induced even under long-day (LD) conditions, resulting in the up-regulation of *FT2a* and *FT5a* expression, which induces flowering (Xia *et al.*, 2012). The *E2* gene is an ortholog of Arabidopsis *GIGANTEA* (*GI*) (Watanabe *et al.*, 2011). GI is a nuclear-localized membrane protein, which interacts with FLAVIN-BINDING, KELCH-REPEAT, F-BOX 1 (FKF1) to up-regulate the expression of *CONSTANS* (*CO*) through degradation of CYCLING DOF FACTOR (CDF) (Huq *et al.*, 2000), and which also activates *FT* expression by directly binding to a *cis*-element (Sawa *et al.*, 2007). Unlike *E1*, *E2* is not involved in NB responses of soybean (Xu *et al.*, 2015), and therefore *E1* and *E2* appear to control flowering time via different pathways. Recently, the maturity gene *E9* was identified as *FT2a*; its late-flowering *e9* allele has a *Ty1/copia*-like retrotransposon inserted in the first intron, which attenuates transcript abundance (Zhao *et al.*, 2016).

Different allelic combinations at the above five loci (*E1*–*E4* and *E9*) produce diverse flowering habits in soybean cultivars (Xu *et al.*, 2013; Kong *et al.*, 2014; Tsubokura *et al.*, 2014; Zhao *et al.*, 2016). Using regression analyses, Tsubokura *et al.* (2014) found that multi-locus genotypes at *E1* to *E4* account for 62–66% of natural variation in flowering time among (mainly Japanese) soybean cultivars. Various allelic combinations at the *E1*, *E3*, and *E4* loci control the absence of or reduced photoperiod sensitivity, which is essential for adaptation to high latitudes, although this trait is also affected by an unknown gene(s) (Xu *et al.*, 2013). Genotyping with functional DNA markers for identified maturity loci has improved our understanding of the relationship between maturity genotypes and flowering habits in various regions at different latitudes and has also uncovered novel genetic variations that affect flowering (Xu *et al.*, 2013; Kong *et al.*, 2014; Tsubokura *et al.*, 2014; Lu *et al.*, 2016).

Many QTLs controlling time to flowering have been reported in soybean (Keim *et al.*, 1990; Mansur *et al.*, 1993; Lee *et al.*, 1996; Orf *et al.*, 1999; Tasma *et al.*, 2001; Yamanaka *et al.*, 2001; Chapman *et al.*, 2003; Wang *et al.*, 2004; Watanabe *et al.*, 2004; Zhang *et al.*, 2004; Funatsuki *et al.*, 2005; Pooprompan *et al.*, 2006; Githiri *et al.*, 2007; Komatsu *et al.*, 2007; Liu *et al.*, 2007; Khan *et al.*, 2008; Liu and Abe, 2010; Cheng *et al.*, 2011; Liu *et al.*, 2011; Yamaguchi *et al.*, 2014; Lu *et al.*, 2016). The molecular dissection of QTLs whose functions remain undetermined is important for better understanding of the molecular mechanisms underlying natural variations of flowering time in soybean, and also for marker-assisted breeding for flowering time.

Here we describe the molecular dissection of a QTL for flowering time detected in two independent crosses between early-maturing soybean cultivars. Fine-mapping and subsequent sequencing and expression analyses have identified *FT5a* as a gene responsible for this QTL.

## Materials and methods

### Plant material

Segregating populations of two soybean crosses, Toyoharuka (TH) × 1532-1 (cross A), and a near-isogenic line (NIL) of Harosoy for *e3* (H-*e3*, PI547716) × Jiagedaqi-02 (J02) (cross B), were used in this study. TH and 1532-1 have the same maturity genotype at the *E2*, *E3*, and *E9* loci (*e2*/*E3*/*e9*), but differ at the *E1* and *E4* loci: TH has the *e1-nl* allele, which lacks the entire *E1* genomic region, and the *e4* allele, whereas 1532-1 has functional *E1* and *E4* alleles (Yamaguchi *et al.*, 2014). J02 is a breeding line developed at the Agricultural Experimental Station at Jiagedaqi, Heilongjiang Province, China. It has the same maturity genotype as H-*e3* at all of the five maturity loci (*e1-as*/*e2*/*e3*/*E4*/*E9*), but unlike H-*e3* it exhibits a reduced photoperiod sensitivity of flowering under incandescent LD conditions (ILD; Saindon *et al.*, 1989), where natural daylength is extended to 20h by using incandescent lamps with low R:FR ratios (Xu *et al.*, 2013). Recombinant inbred lines (RILs) for each of the two crosses were developed with the single seed descent method. The RIL population consisted of 99 lines homozygous for *e1-nl* and 62 lines homozygous for *E1* in cross A and 79 lines in cross B. NILs for the early-flowering (*ef*) and late-flowering (*lf*) alleles at a QTL were developed from the progenies of F_6_ or F_7_ plants used for fine-mapping in cross A (NILs #46 and #64) and from the progeny of an F_6_ plant in cross B (NIL #8).

### Field experiment

Flowering time in the RIL population of cross A was evaluated at the Tokachi Agricultural Experiment Station (42°91′N, 143°05′E) in 2010 (for F_5_) and 2011 (for F_5:6_) (Yamaguchi *et al.*, 2014). The progeny test was carried out in an experimental field at Hokkaido University, Sapporo (43°07′N, 141°35′E) in 2013 and 2014. Segregation in cross B was examined in an ILD field at Hokkaido University where natural daylength was extended to 20h by using incandescent lamps set at 2 m height for F_2_ in 2012 and for the RIL population (F_6_) in 2015. In the field experiments at Hokkaido University, seeds were sown in paper pots in a plastic greenhouse (cross A) or in the ILD field (cross B), and 10 d later the seedlings were transplanted into the field. Sowing dates were 1 June in 2012, 10 June in 2013, 29 May in 2014, and 22 May in 2015. The date of the first flower appearance (R1; Fehr *et al.*, 1971) was recorded individually and expressed as the number of days after sowing (DAS).

### DNA marker analysis

Simple sequence repeat (SSR) markers developed by Song *et al.* (2004, 2010), Hisano *et al.* (2007), and Kong *et al.* (2010), and those developed in this study based on the Williams 82 genomic sequence (Schmutz *et al.*, 2010; Gmax v. 2.0, https://phytozome.jgi.doe.gov/pz/portal.html#!info?alias=Org_Gmax) were used for association analysis and linkage mapping. Primers for SSRs developed in this study (SSR-J1 to J5) are listed in Supplementary Table S1 at *JXB* online. DNA extraction and SSR marker analysis were performed as described previously (Kong *et al.*, 2014; Zhao *et al.*, 2016). In addition, DNA markers (FT5a-Pro-indel and FT5a-3′ UTR-indel) were developed to detect insertion/deletions (indels) in the promoter region and the 3′ untranslated region (UTR) of *FT5a*; the amplified products were separated in 1% or 2% agarose gel, stained with ethidium bromide, and visualized under UV light.

### Association and QTL analyses

A linkage map for RILs homozygous for *e1-nl* (cross A) constructed by Yamaguchi *et al.* (2014) was used for QTL analysis; it contained 127 markers and covered 2023 cM. We added six SSRs to fine-map the QTL for flowering time. In cross B, we first tested associations of 100 SSR markers with flowering time by one-way analysis of variance for selected early-flowering and late-flowering F_2_ plants, and then constructed linkage maps for SSR markers significantly associated with flowering time using the whole population. The construction of linkage maps and QTL analyses were performed with JoinMap 4.1 (Van Ooijen, 2011) and MapQTL ver.5 (Van Ooijen, 2004), respectively.

### Fine-mapping

We genotyped seven SSR markers flanking the GMES5027 in the progeny of RILs #46 (*n*=96) and #64 (*n*=27) of cross A, and detected four recombinant plants. Based on the segregation patterns in the progeny, we estimated the genotypes at the QTL for four recombinants and 10 non-recombinant control plants, and compared them with graphical genotypes constructed by using SSR markers. BLAST was used to search for homology.

### Sequence analysis

The *FT5a* genomic region (4858bp) from 3.0kb upstream from the start codon to the end of the 3′ UTR was sequenced for the four parents. PCR was performed with *ExTaq* polymerase (TaKaRa) with total DNA as a template. The amplified fragments were ligated into the pGEM T-Easy vector (Promega), and cloned into *E. coli* JM109 Competent Cells (TaKaRa). Purified plasmids were used as templates for forward and reverse sequencing reactions by using a BigDye Terminator v. 3.1 Cycle Sequencing kit, and sequenced with an ABI PRISM 3100 Avant Genetic Analyzer (both from Applied Biosystems) in accordance with the manufacturer’s instructions. Plant *cis*-acting regulatory DNA elements (PLACE; Higo *et al.*, 1999) analysis and DNA pattern search (http://www.geneinfinity.org/sms/sms_DNApatterns.html) were carried out to detect possible *cis*-elements in the *FT5a* genomic region. Sequencing primers are listed in Supplementary Table S1. *FT5a* genomic sequences of TH, 1532-1, H-*e3*, and J02 can be found in the GenBank/EMBL/DDBJ data libraries under the accession numbers LC128590, LC128591, LC128592, and LC128593, respectively.

### Expression analysis

NILs for the *ef* and *lf* alleles at the QTL detected were grown in a greenhouse at an average temperature of 24 °C; natural daylength (<12h in November and December) was extended to 20h by using incandescent lamps. Fully developed trifoliate leaves were sampled individually at Zeitgeber time 3 at 15, 25, and 35 d after emergence, immediately frozen in liquid N_2_, and stored at −80 °C. Total RNA was isolated from frozen leaves by using TRIzol Reagent (Invitrogen). DNase I (TaKaRa) was used to remove genomic DNA. cDNA was synthesized from 1 µg of total RNA using an oligo (dT) 20 primer or a random primer cocktail (TaKaRa). Transcript levels of *FT5a*, *FT2a*, and *E1* were determined by quantitative real-time PCR (qRT-PCR). Each qRT-PCR mixture (20 µl) contained 0.05 µl of the cDNA synthesis reaction, 5 µl of 1.2 µM primer premix, and 10 µl SYBR Premix *ExTaq* Perfect Real Time (TaKaRa). A CFX96 Real-Time System (Bio-Rad) was used. The PCR cycling conditions were 95 °C for 3min followed by 40 cycles of 95 °C for 10s, 58 °C for 30s, 72 °C for 20s, and 78 °C for 2s. Fluorescence was quantified before and after the incubation at 78 °C to monitor the formation of primer dimers. The mRNA for β*-tubulin* was used as an internal control. A reaction mixture without reverse transcriptase was also used as a control to confirm the absence of genomic DNA contamination. For each transcript, amplification of a single DNA fragment was confirmed by melting curve analysis and gel electrophoresis of the PCR products. Averages and standard errors of relative expression levels were calculated from qRT-PCR results for three independent plants. Primers used in expression analyses are listed in Supplementary Table S2.

### RACE analysis

3′ RACE was performed to determine the 3′ UTR sequences of the *ef* and *lf* alleles using the SMARTer RACE cDNA Amplification Kit (Clontech). The primer 5′-GCCCTAGGGTTACTGTTGGTGGTGAA-3′ was designed based on the Williams 82 sequence. The cDNAs from NILs for *ef* and *lf* alleles were used as templates, and the amplified products were cloned and sequenced.

### Single-nucleotide polymorphism (SNP) calling

SNPs were called from the re-sequencing data of 302 worldwide cultivated and wild soybean collections (Zhou *et al.*, 2015) and 137 early-maturing landraces and improved cultivars developed in northeast China (Liu *et al.*, unpublished data). Paired-end re-sequencing reads were mapped to the Williams 82 soybean reference genome (Gmax_275_Wm82.a2.v1; Schmutz *et al.*, 2010) with the Burrows–Wheeler Aligner software (v0.7.10) (Li and Durbin, 2009) using the default parameters. The SAMtools software (v0.1.19) (Li *et al.*, 2009) was used to convert mapping results into BAM (binary alignment/map) format and then to sort the BAM files by the chromosomal position of the SNP. Duplicated reads were filtered with the Picard package (v1.90) (http://broadinstitute.github.io/picard). The GATK software (v3.0-0-g6bad1c6) (McKenna *et al.*, 2010) was used to realign the reads around indels and produce a realigned BAM file for each accession as follows: the RealignerTargetCreator tool was used to identify regions where realignment was needed, and then the IndelRealigner tool was used to realign these regions. SNPs were called at a population level with SAMtools. SNPs with quality scores <40 were discarded. Haplotype networks were constructed using SNPs with frequencies of rare variants of 4% or more.

## Results

### Segregation of flowering time

**Fig. 1.**
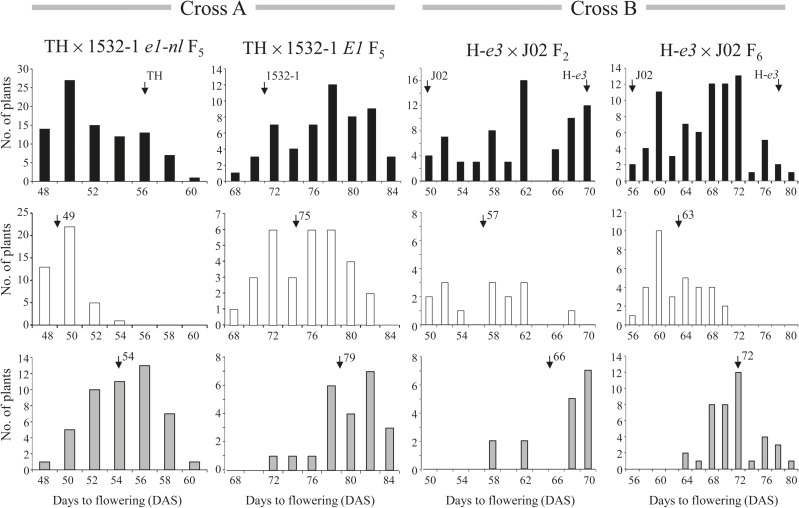
Segregation of flowering time in the progeny of two crosses, Toyoharuka (TH) × 1532-1 (Cross A) and Harosoy near-isogenic lines for *e3* (H-*e3*) × Jiagedaqi-02 (J02) (Cross B). Frequency distributions of days to flowering (DTF) are presented for the whole population (closed bars), plants or lines homozygous for the *ef* allele (open bars), and those homozygous for the *lf* allele (grey bars) at *qDTF-J*. In cross A, recombinant inbred lines (F_5_) were classified into lines homozygous for *e1-nl* and *E1* alleles. Flowering time was recorded as the date of the appearance of the first flower (R1). Arrows indicate the average flowering times of parents and lines with the *ef* or *lf* alleles. The numbers near the arrows indicate the mean values of flowering time in each genotypic class (*ef* or *lf*). DAS, days after sowing.

Frequency distributions of flowering time in the segregating populations of the two crosses are presented in Fig. 1. The RILs (F_5_) of cross A exhibited a broad distribution from 47 to 84 DAS. The observed variation was mostly accounted for by the *E1* genotypes; RILs homozygous for *e1-nl* flowered at 47 to 60 DAS, whereas those homozygous for *E1* flowered on average 20 d later. The flowering times of F_6_ plants were significantly correlated (*r*=0.84, *P*<0.001) with those of their parents (F_5_), indicating that flowering times in RILs were stable between 2010 and 2011.

The segregation of flowering time in cross B was evaluated under ILD conditions. The F_2_ and F_6_ RIL populations showed a continuous distribution, with flowering times intermediate between those of the parents, J02 and H-*e3*. The frequency of plants that flowered at almost the same time as H-*e3* was 16% in F_2_ and 10% in F_6_, and was lower than expected from monogenic inheritance (25% in F_2_ and approximately 50% in F_6_), suggesting that at least two genes contribute to the difference in photoperiod sensitivities between H-*e3* and J02.

### QTL analysis for flowering time

**Fig. 2.**
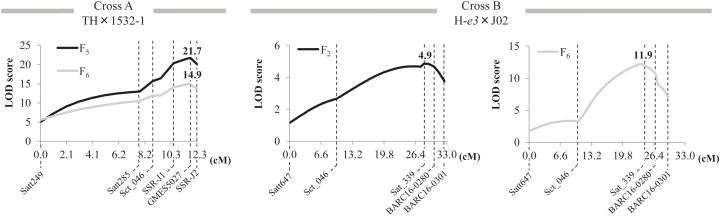
Logarithm (base 10) of odds (LOD) score plots of *qDTF-J1* and *qDTF-J2*, the QTLs for flowering time in linkage group J (Chr. 16), in Toyoharuka (TH) × 1532-1 (Cross A) and Harosoy near-isogenic line for *e3* (H-*e3*) × Jiagedaqi-02 (J02) (Cross B), respectively.

Yamaguchi *et al.* (2014) identified a QTL for days to flowering (DTF) in linkage group J (Chromosome16, here tentatively designated as *qDTF-J1*) in 99 RILs of cross A homozygous for *e1-nl*. To map *qDTF-J1* more precisely, we added two SSR markers (SSR-J1 and SSR-J2) and recalculated the logarithm (base 10) of odds (LOD) scores (Fig. 2). The highest LOD scores (21.7 and 14.9) were detected at GMES5027, the marker that was also detected in both F_5_ (2012) and F_6_ (2013) populations by Yamaguchi *et al.* (2014). This QTL accounted for 55% of the total phenotypic variance in flowering time in F_5_ and for 36% in F_6_. The additive effect of the TH allele was 2.4 d in the F_5_ population and 1.4 d in the F_6_ population.

To detect QTLs for flowering time in cross B, we tested the association between marker genotypes and flowering times for selected plants, eight early-flowering and eight late-flowering. Of the 100 SSR markers tested, eight markers showed significant associations, and linkage maps of their flanking regions were constructed for QTL analysis. By using linkage maps covering 408 cM, the major QTL was detected in linkage group J (Chr. 16), which had the highest LOD score, 4.9 (Fig. 2), and two minor QTLs were detected in linkage groups G (Chr. 18) and O (Chr. 10), which had LOD scores of 2.7 and 1.9, respectively (Supplementary Fig. S1). The QTL in linkage group J (tentatively designated as *qDTF-J2*) accounted for 27% of the total phenotypic variance in the F_2_ population. The tagging marker Sat_339 was located near GMES5027 (Hwang *et al.*, 2009), indicating that *qDTF-J2* may be identical to or located close to *qDTF-J1*. *qDTF-J2* was further confirmed in the F_6_ RIL population; the LOD score was 11.9 (Fig. 2), and it accounted for 51% of the total variance in flowering time. The additive effect of the H-*e3* allele was 4.9 d in the F_2_ population and 4.1 d in the F_6_ population.

### Fine-mapping of qDTF-J1

**Fig. 3.**
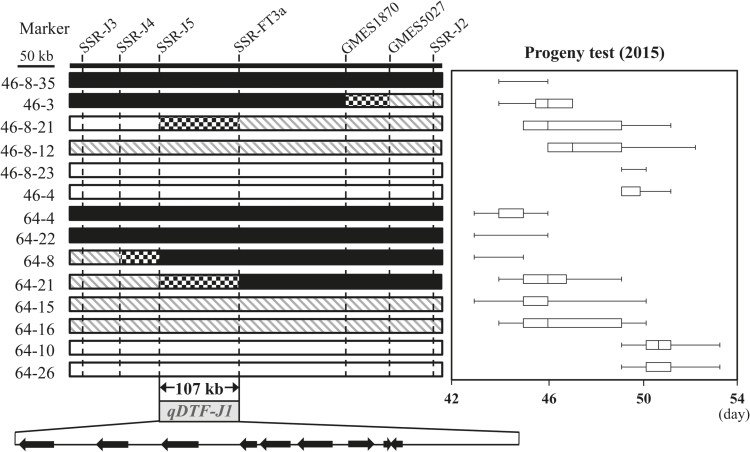
Fine-mapping of *qDTF-J1* and annotated genes in the delimited genomic region. Four recombinants, two (#46-3 and #46-8-21) from RIL #46 and two (#64-8 and #64-21) from RIL #64, and ten non-recombinant control plants were genotyped at seven SSRs. The genotype at *qDTF-J1* was estimated by progeny testing (right panel). The segregation of flowering time (DAS, days after sowing) is indicated in a box-plot format with ranges (horizontal lines), interquartile ranges (boxes), and medians (vertical lines). Closed bars, regions homozygous for the *ef* allele; open bars, homozygous for the *lf* allele; hatched bars, heterozygous; chequered bars, regions where recombinations occurred. Nine open reading frames (arrows) are predicted in a genomic region of 107-kb delimited by SSR-J5 and SSR-FT3a.

To narrow down the genomic position of *qDTF-J1*, we genotyped seven SSR markers flanking GMES5027 in the progeny of RILs #46 (*n*=96) and #64 (*n*=27), and detected four recombinant plants. Based on the segregation patterns in the progeny, we estimated the genotypes at *qDTF-J1* for four recombinants and ten non-recombinant control plants, and compared them with graphical genotypes constructed by using SSR markers (Fig. 3). The recombinant plant #46-3, which was homozygous for the 1532-1 allele in the region from SSR-J3 to GMES1870 but heterozygous in the region from GMES5027 to SSR-J2, flowered as early as plant #46-8-35, which was homozygous for the 1532-1 allele in the whole region. Plant #46-8-21, which was homozygous for the TH allele in the region from SSR-J3 to SSR-J5 but heterozygous in the region from SSR-FT3a to SSR-J2, segregated for flowering time similar to the heterozygous plant #46-8-12. The recombinant plant #64-8, which was heterozygous in the region from SSR-J3 to SSR-J4 but homozygous for the 1532-1 allele in the region from SSR-J5 to SSR-J2, flowered as early as plants #64-4 and #64-22, which were homozygous for the 1532-1 allele. Plant #64-21, which was heterozygous in the region from SSR-J3 to SSR-J5 but homozygous for the 1532-1 allele in the region from SSR-FT3a to SSR-J2, segregated similar to heterozygous plants #64-15 and #64-16. Based on these results, we delimited the QTL to a 107-kb region between SSR-J5 and SSR-FT3a. According to the Williams 82 genome sequence (Schmutz *et al.*, 2010), nine genes are annotated in this region: four genes for apyrase proteins (Glyma.16G043300, Glyma.16G043400, Glyma.16G043500, Glyma.16G043700) and one gene each for tetratricopeptide repeat-like superfamily protein (Glyma.16G043600), an aquaporin-like superfamily protein (Glyma.16G043800), a transmembrane protein of unknown function with a DUF106 domain (Glyma.16G043900), FT5a (Glyma.16G044100), and an unannotated protein (Glyma.16G044000) (Table 1). BLAST searching revealed that Glyma.16G044000 had 86% amino acid sequence identity with *Glycine soja* NAD(P)H-quinone oxidoreductase subunit O (European Nucleotide Archive accession number KHN09611). According to the analysis of transcriptional networks that contribute to floral initiation under inductive SD (Wong *et al.*, 2013), the expression of all of the annotated genes except Glyma.16G044100 (*FT5a*) was not up-regulated, whereas *FT5a* expression was up-regulated shortly after SD induction. Because *FT5a* is a functional *FT* ortholog and promotes flowering of soybean under non-inductive conditions when ectopically expressed (Nan *et al.*, 2014; Guo *et al.*, 2015), *FT5a* was the most likely candidate for *qDTF-J1*. *FT5a* is linked in tandem to *FT3a* (Kong *et al.*, 2010). The genotypes at SSR-FT3a, which targets the SSR in the first intron of *FT3a* (Kong *et al.*, 2010), were inconsistent with the estimated genotype at *qDTF-J1* (Plant #64-21).

**Table 1.** Annotation of the genes in the delimited genomic region for *qDTF-J1* by fine-mapping.

Gmax 2.0 primary protein ID	Annotation
Glyma.16G043300	Apyrase 2 (ATAPY2, APY2)
Glyma.16G043400	Apyrase 2 (ATAPY2, APY2)
Glyma.16G043500	Apyrase 2 (ATAPY2, APY2)
Glyma.16G043600	Tetratricopeptide repeat like superfamily protein
Glyma.16G043700	Apyrase 2 (ATAPY2, APY2)
Glyma.16G043800	Aquaporin-like superfamily protein (SIP1;2)
Glyma.16G043900	Protein of unknown function DUF106, transmembrane
Glyma.16G044000	Not annotated
Glyma.16G044100	Phosphatidylethanolamine-binding protein (FT, PEBP)

### Sequence analysis of *FT5a*

**Fig. 4.**
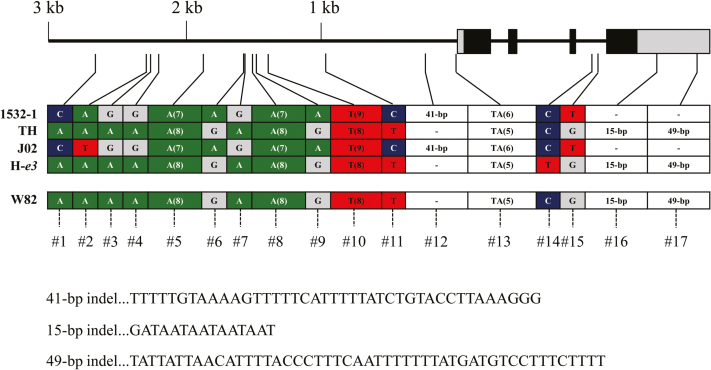
DNA polymorphisms in the *FT5a* genomic region among four soybean cultivars and the Williams 82 reference sequence. Gray boxes, UTR; closed boxes, exons. Black bars indicate the promoter and introns. TH, Toyoharuka; J02, Jiagedaqi-02; W82, Williams 82; H-*e3*, near-isogenic line of Harosoy for *e3*. This figure is available in color at *JXB* online.

We sequenced the genomic region of *FT5a* (4858bp) in the parents of the two crosses from 3.0kb upstream from the start codon to the end of the 3′ UTR. In all four parents, the sequences of coding regions were identical to that of Williams 82. There were a total of 17 polymorphisms among the four parents: eight SNPs (#1, 2, 3, 4, 6, 7, 9, and 11), one indel (#12), and four SSRs (#5, 8, 10, and 13) in the promoter region; two SNPs (#14 and 15) in the third intron; and two indels (#16 and 17) in the 3′ UTR (Fig. 4). The two indels (15bp and 49bp) in the 3′ UTR were confirmed by 3′ RACE. Because of these two deletions, the 3′ UTR of *FT5a* from 1532-1 (472bp) was 64bp shorter than that from TH.

1532-1 and J02 shared the same polymorphisms except for one SNP (#2) in the promoter. In contrast, TH and H-*e3* differed from 1532-1 and J02 by the same eight SNPs, three indels, and four SSRs. The Williams 82 sequence (*Glyma.16G044100*) differed from those of TH and H-*e3* in one and two SNPs, respectively. Although fine-mapping was performed only for *qDTF-J1*, *qDTF-J2* is most likely identical to *qDTF-J1*, because both QTLs were mapped at adjacent SSRs, and the DNA polymorphisms in the candidate gene were common between the two crosses. The two QTLs are renamed hereafter as *qDTF-J* with the early-flowering (*ef*) and late-flowering (*lf*) alleles.

### Confirmation of the effect of *qDTF-J* on flowering time

We confirmed the association between the genotype at *qDTF-J* and flowering time by using the progeny of RILs heterozygous for *qDTF-J* and derived NILs for the *ef* and *lf* alleles. A DNA marker to detect the indel in the *FT5a* promoter was used to genotype *qDTF-J*. In the progeny of four *e1-nl*-RILs heterozygous at *qDTF-J* in cross A (#46, 47, 64, and 97), plants homozygous for the *ef* allele from 1532-1 flowered on average 2.3 to 4.1 d earlier than those homozygous for the *lf* allele from TH in the field condition (Table 2). Differences in average flowering times between plants homozygous for the marker genotypes were statistically significant for all RILs except #47. The association was further confirmed in the progeny (F_8_) of three heterozygous plants selected from RILs #47 and #97. *qDTF-J* thus had a stable (although a relatively small) effect on flowering time. In all segregating families (F_7_ and F_8_), average flowering times of heterozygous plants were almost the same or slightly later than those of plants homozygous for the *ef* allele, but earlier than those of plants homozygous for the *lf* allele, suggesting that the *ef* allele at *qDTF-J* behaved as a dominant or partially dominant allele.

**Table 2.** Associated segregation of genotypes at a DNA marker FT5a-Pro-indel with flowering time in field conditions.

Family	No. of plants	Flowering time (mean ± SD)			One-way ANOVA	
		AA	AB	BB	*F*-value	*P*
F_7_ (2013)						
L46	21	42.3±1.0	40.3±1.5	39.9±1.3	4.1	0.034
L47	24	41.3±1.3	39.3±2.0	39.0±1.0	3.5	0.052
L64	27	40.8±0.7	38.8±1.3	36.7±1.6	18.6	<0.001
L97	28	43.1±1.3	41.2±1.3	39.0±2.2	11.4	<0.001
F_8_ (2014)						
L47-8	67	48.5±1.8	44.4±1.5	43.4±0.8	62.1	<0.001
L97-2	36	47.3±1.6	44.1±0.9	43.5±0.8	31.4	<0.001
L97-5	35	46.2±2.6	43.8±1.0	43.3±0.7	10.1	<0.001

A and B indicate alleles from Toyoharuka and 1532-1, respectively.

Flowering time indicates number of days after sowing

**Fig. 5.**
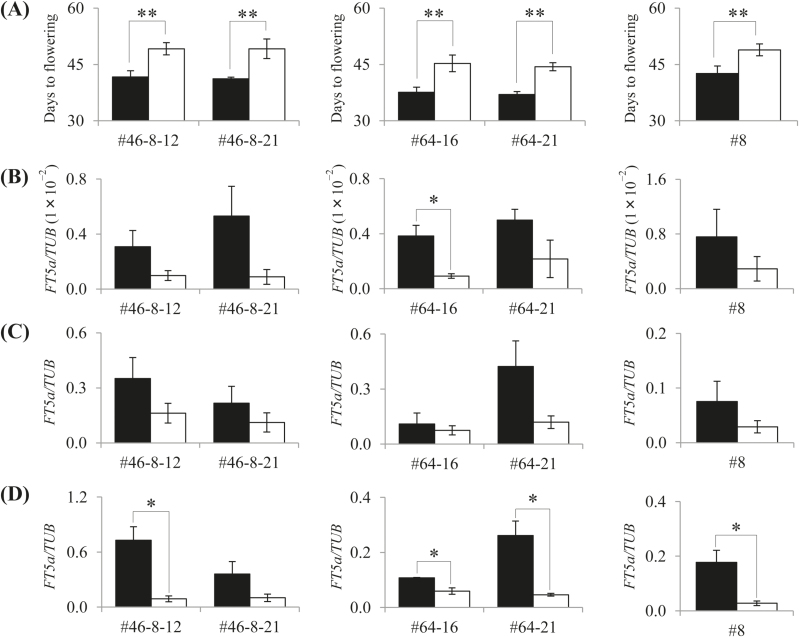
Flowering time and *FT5a* expression in near-isogenic lines for the *ef* (closed bars) and *lf* (open bars) alleles at *qDTF-J*. (A) Days to flowering after emergence (DAE) under LD conditions (20h light/4h dark). Error bars show standard deviation (*n*=10). (B–D) Relative levels of *FT5a* mRNA at (B) 15 DAE, (C) 25 DAE, and (D) 35 DAE. Values are given relative to β*-tubulin* (TUB) transcript levels. Error bars show standard error of the mean of three biological replicates (independent plants). * *P*<0.05 and ** *P*<0.001 (Student’s *t* test).

The flowering time in the LD condition was also significantly different (*P*<0.001) between the *ef* and *lf* alleles in all sets of NILs tested in cross A: the NILs for *ef* flowered 8 to 10 d earlier than those for *lf* (Fig. 5A). In the progeny of RIL #8 from cross B, plants homozygous for the *ef* allele from J02 flowered on average 8 d earlier than plants homozygous for the *lf* allele from H-*e3* (Fig. 5A).

### Association between flowering time and *FT5a* expression levels

To determine whether the difference in flowering times between plants carrying different alleles at *qDTF-J* could be explained by different *FT5a* transcript levels, we compared the *FT5a* transcript levels between the NILs. The expression levels of *FT5a* were higher in NILs for the *ef* allele than in those for the *lf* allele at all three time points tested (Fig. 5B–D). Some of these differences were statistically significant, in particular at 35 d after emergence. Therefore, early flowering in the NILs was associated with higher expression levels of *FT5a*. We also analyzed the expression of eight annotated genes other than *FT5a* in the 107-kb genomic region. As expected, their expression patterns varied among the three sets of NILs (Supplementary Fig. S2)

We also assayed the *FT2a* transcript abundance in NILs for the *ef* and *lf* alleles to evaluate whether it was associated with the difference in flowering times. *FT2a* expression was very low in the NILs developed from cross A (Supplementary Fig. S3) because both parents had the *e9* allele, in which *SORE-1* inserted in the first intron attenuates *FT2a* expression (Zhao *et al.*, 2016). Low *FT2a* expression was also found in NILs developed from cross B; the low expression was probably due to *E1* expression (Supplementary Fig. S3). There was no difference in *FT2a* expression levels between NILs for the *ef* and *lf* alleles, suggesting that the difference in *FT5a* expression most likely contributed to the differences in flowering times between NILs.

### PLACE analysis and DNA pattern search for *cis* elements

We compared the distribution of *cis* elements in the *FT5a* genomic region between the *ef* and *lf* alleles with a focus on the known *cis* elements in Arabidopsis *FT* recognized by various transcriptional factors, such as the CCAAT box for nuclear factor Y (NF-Y), CO-responsive elements CORE1 and CORE2, the CArG box for MADS-box proteins, the E box for cryptochrome 2-cryptochrome-interacting basic helix-loop-helix (CIB) complex, and the Dof-binding site (AAAG) for CYCLING DOF FACTOR (CDF) (Andrés and Coupland, 2012; Song *et al.*, 2015). By using PLACE (Higo *et al.*, 1999) and DNA pattern search, we found that these *cis* elements were similar in both alleles, except for three indels. An insertion of 41bp (#12; Fig. 4) in the promoter of the *ef* allele contained one CArG box and two Dof elements, whereas two insertions in the *lf* allele (#16 and 17) contained three Dof elements. In the *ef* allele, deletion #16 generated a novel E box. In addition, the insertion in the *ef* allele contained an I box core motif (GATAA), a sequence conserved upstream of light-regulated genes, which activates their transcription in response to light signals (Terzaghi and Cashmore, 1995; Martínez-Hernández *et al.*, 2002; López-Ochoa *et al.*, 2007). The difference in *cis* elements in the indels may be responsible for the differences between the alleles in *FT5a* expression levels.

### DNA marker analysis and SNP calling

**Fig. 6.**
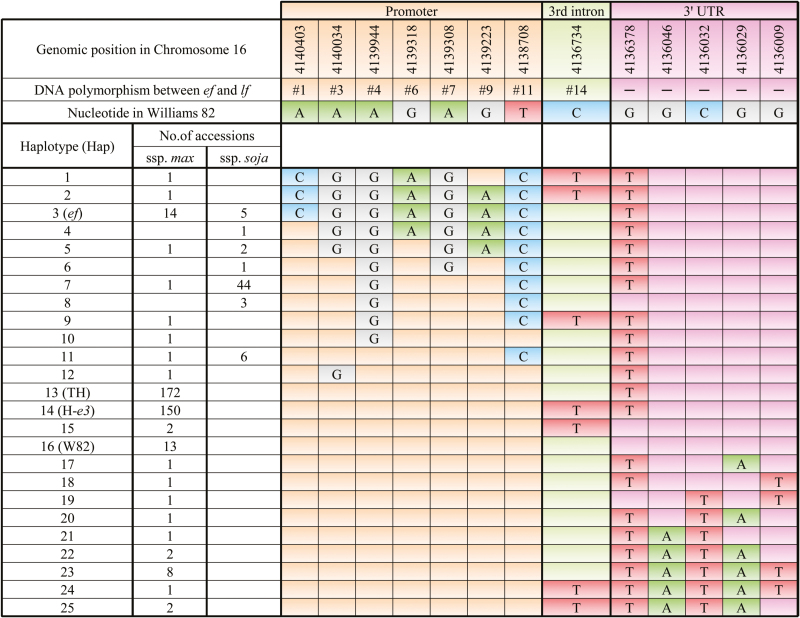
Haplotypes identified with 13 SNPs detected in the *FT5a* genomic region in 439 soybean accessions. Blanks mean the same nucleotide as in Williams 82. TH, Toyoharuka; J02, Jiagedaqi-02; W82, Williams 82; H-*e3*, near-isogenic line of Harosoy for *e3.* This figure is available in color at *JXB* online.

Genetic materials with intermediate combinations of DNA polymorphisms between the *ef* and *lf* alleles may be useful to determine which polymorphisms are responsible for different expression levels. We first surveyed the 41-bp insertion in the promoter and two deletions (15bp and 49bp) in the 3′ UTR in 50 early-maturing accessions (Xu *et al.*, 2013). Ten accessions had the same combination of polymorphisms as the *ef* allele; there were no accessions with the insertion only or the deletions only (Table S3). We then carried out SNP calling from re-sequencing data of 439 soybean accessions. A total of 114 polymorphisms, which included 94 SNPs, seven indels of <10bp, and 13 SSRs, were detected. Using 13 SNPs with frequencies of rare variants of 4% or more in the cultivated soybean population, we identified 22 haplotypes in the 377 cultivated accessions and seven haplotypes in the 62 wild accessions (Fig. 6). The *ef* alleles from 1532-1 and J02 corresponded to haplotype Hap3, whereas the *lf* alleles corresponded to haplotypes Hap13 in TH and Hap14 in H-*e3*. Hap13 (frequency, 46%) and Hap14 (40%) were most common, whereas Hap3 (4%) was rarely observed; all of the Hap3 accessions originated in northern Japan and northern China. Haplotypes Hap17 to Hap25 differed from Hap13 and Hap14 in SNPs in the 3′ UTR. Interestingly, there were almost no intermediate haplotypes between Hap3 (*ef*) and Hap13 and Hap14 (*lf*) in the cultivated soybean; only five accessions shared one or two of seven diagnostic SNPs of the *ef* allele. There were no polymorphisms in the promoter among 13 haplotypes including the two most common haplotypes. Wild soybean shared four haplotypes, Hap3, Hap5, Hap7, and Hap11, with cultivated soybean, but the haplotypes most common in cultivated accessions (Hap13 and Hap14) were not found in wild accessions. Taken together, these data show that the *ef* allele is a rare haplotype distinct from the haplotypes most common in the cultivated soybean population; it is also present in the wild soybean population.

Because the three indels between the *ef* and *lf* alleles could not be identified with certainty in the re-sequencing data, we used DNA markers to analyze eight northeastern Chinese accessions with Hap3 or Hap5 for the presence of these indels. Seven accessions with Hap3 had the same combination of indels as the *ef* allele, whereas the accession with Hap5 had the same combination as the *lf* allele.

We also compared flowering times between accessions carrying the *ef* or *lf* alleles among 50 early-flowering accessions with known maturity genotypes (Xu *et al.*, 2013). The accessions with the *ef* allele flowered earlier than those with the *lf* allele among the accessions with the maturity genotype of *e1*/*e2*/*e3*/*E4* or *e1-as*/*e2*/*e3*/*E4*, although only one accession had the *ef* allele among those with each of the two genotypes (Supplementary Table S4). A further study with more accessions is needed to confirm the association detected in each of the two genotypic classes.

### *qDTF-J* controls flowering time in the *E1* genetic background

The abundance of the *FT5a* transcript depends on the genotype at the *E1* locus (Xu *et al.*, 2015). To determine whether the effect of *qDTF-J* on flowering time depends on the genotype at the *E1* locus, we assessed the association between the genotype at *qDTF-J* and flowering time in the *E1*-RIL population of cross A. The ranges of flowering time were similar (spanning ~13 d) in the *e1-nl*-RIL and *E1*-RIL F_5_ populations (Fig. 1). In both populations, RILs homozygous for the *ef* allele flowered earlier (on average by 4 d) than those homozygous for the *lf* allele; the difference in average flowering time between the *FT5a* genotypes was highly significant (*P*<0.001) in both *E1* genotypes. A similar effect of the *FT5a* alleles was also detected in cross B (Fig. 1). Therefore, *qDTF-J* controlled flowering time independently of the *E1* genotype.

## Discussion

### *FT5a* is a candidate for *qDTF-J*

In this study, we describe molecular dissection of a QTL for flowering time under LD conditions, which was detected in linkage group J (Chr. 16) in two independent crosses between early-maturing soybean cultivars. Fine-mapping delimited the QTL to a 107-kb region that contained nine annotated genes, including *FT5a*, a soybean ortholog of *FT*. Sequencing of the *FT5a* genomic region revealed that, despite the identical coding sequences, parents carrying the *ef* allele at the QTL differed by a total of 15 DNA polymorphisms from parents carrying the *lf* allele. In both crosses, *FT5a* expression levels were higher in NILs for the *ef* allele than in NILs for the *lf* allele. Taken together, the data indicate that the QTLs detected in the two crosses were identical, and different expression of *FT5a* was the cause of the difference in flowering time.

*FT2a* and *FT5a* appear to play major roles as florigens and redundantly initiate flowering in soybean (Kong *et al.*, 2010; Fan *et al.*, 2014; Guo *et al.*, 2015). The overexpression of *FT2a* and *FT5a* driven by the 35S promoter promotes flowering of soybean plants under non-inductive conditions (Sun *et al.*, 2011; Nan *et al.*, 2014; Guo *et al.*, 2015). Recently, *FT2a* was determined to be identical to the maturity gene *E9* (Zhao *et al.*, 2016). The DNA polymorphisms detected in this study in the *FT5a* genomic region probably contribute to the natural variation of flowering time in soybean by affecting *FT5a* transcript levels.

Various transcription factors bind to the *cis* elements of *FT* to control its expression in Arabidopsis (reviewed by Andrés and Coupland, 2012; Song *et al.*, 2015). In Arabidopsis, the zinc-finger transcriptional regulator CO integrates the circadian rhythm and light signals to directly interact with the *FT* promoter and activate *FT* transcription (Putterill *et al.*, 1995; Imaizumi *et al.*, 2003). It directly binds to CORE1 and CORE2, which have the consensus sequence TGTG(N2-3)ATG (Tiwari *et al.*, 2010). The NF-Y complex interacts with CO and binds to the distal enhancer element of *FT* (CCAAT) to recruit CO to proximal *cis* regulatory elements in the *FT* promoter (Cao *et al.*, 2014). Furthermore, MADS-box proteins, such as FLOWERING LOCUS C (FLC) and SHORT VEGETATIVE PHASE (SVP), bind to the CArG boxes in the promoter and introns of *FT* (Helliwell *et al.*, 2006; Lee *et al.*, 2007; Li *et al.*, 2008; Deng *et al.*, 2011; Tao *et al.*, 2012; Balanzà *et al.*, 2014; Mateos *et al.*, 2015). CDFs and CIBs bind to the Dof element and the E box, respectively (Song *et al.*, 2015). We found one CArG and two Dof elements in the 41-bp insertion in the promoter of the *ef* allele and three Dof elements in two insertions in the *lf* allele. Additionally, the insertion in the *ef* allele contained an I box, and an E box was generated by a 49-bp deletion (#17) in 3′ UTR.

Different levels of *FT5a* mRNA might also be explained by differences in its stability caused by polymorphisms in the 3′ UTR, which may affect miRNA-binding sites or mRNA conformation (DeRidder *et al.*, 2012). In plants, most experimentally verified miRNA target sites are located in coding regions, with only a few in 5′ UTRs or 3′ UTRs, or in non-coding RNAs (Allen *et al.*, 2005; Addo-Quaye *et al.*, 2008; German *et al.*, 2008). We surveyed the miRNA target sites in the 3′ UTR of *FT5a* by using the TAPIR program (Bonnet *et al.*, 2010), but could not detect any target sites in the two *qDTF-J* alleles. Further studies are needed to determine not only whether the *cis* elements detected in the insertions in the promoter and 3′ UTR have any effect on *FT5a* expression but also whether deletions in the 3′ UTR influence *FT5a* mRNA stability.

### Molecular diversity of *FT5a* and the origin of the *ef* allele

We found that the *ef* allele corresponded to a rare haplotype (Hap3) that was distinct from the common haplotypes (Hap13 and Hap14) corresponding to the *lf* alleles from TH and H-*e3*; the accessions with Hap3 had the same combination of three indels as the *ef* allele. Because the accession with Hap5, which differed by two SNPs from Hap3, had the same combination of indels as the *lf* allele, the 41-bp insertion in the promoter and 15-bp and 49-bp deletions in the 3′ UTR may be characteristics of Hap3.

Because of its low frequency, the *ef* allele may not have been a major contributor to the natural variation of flowering time in cultivated soybean. Rather, it may have adaptive significance in some environments, in particular at high latitudes where early-flowering genotypes are desirable. The distribution of SNPs was discontinuous between Hap3 (*ef* allele) and common haplotypes (*lf* allele), suggesting that the *ef* allele did not originate from the *lf* allele via the accumulation of mutations. Because Hap3 was also observed in the wild soybean population, the *ef* allele might have been introgressed from wild soybean during domestication and/or subsequent genetic diversification.

### *qDTF-J* controls flowering time independently of the PHYA–E1 pathway

The *E1* locus produces the most marked effect on the flowering time of soybean (Bernard, 1971; Upadhyay *et al.*, 1994; Cober *et al.*, 2001; Watanabe *et al.*, 2004; Tsubokura *et al.*, 2014). It has multiple alleles, including the functional *E1* allele; a leaky *e1-as* allele (traditionally designated *e1*); and dysfunctional alleles, such as *e1-nl*, *e1-fs*, and *e1-b3a* (Xia *et al.*, 2012; Zhai *et al.*, 2015). Although *E1* overexpression strongly inhibits the expression of both *FT2a* and *FT5a* (Xia *et al.*, 2012), *E1* alleles regulate the expression of *FT2a* and *FT5a* differently. The *E1* and *e1-as* alleles inhibit *FT2a* expression similarly, whereas the inhibitory effect of the *E1* allele on *FT5a* expression is stronger than those of *e1-as* and *e1-nl*, suggesting that the *E1* locus controls *FT5a* in a more direct way than it controls *FT2a* (Kong *et al.*, 2010; Xu *et al.*, 2015).

The effects of *qDTF-J* on flowering time, however, were detected in the genetic backgrounds of both *e1-nl* and *E1* (Fig. 1). This is in sharp contrast to the effect of *e9*, a leaky *FT2a* allele, which is expressed at low levels: the effect of *e9* on flowering is detectable only in the *e1-nl* background and not in the *E1* background (Kong *et al.*, 2014; Lu *et al.*, 2016). The DNA polymorphisms that determine the difference in expression between the *ef* and *lf* alleles may not be the same as those involved in the PHYA–E1 pathway, a major controller of flowering in soybean (Xu *et al.*, 2015).

The effect of *qDTF-J* was also detected in the progeny of the cross between H-*e3* and J02, both of which have the *e1-as* allele. J02 has weak photoperiod sensitivity to ILD, although it has the same genotypes at the maturity loci *E1*–*E4* as the Harosoy NIL for *e3*; the latter is photoperiod-sensitive under ILD (Xu *et al.*, 2013). This weak sensitivity of J02 is controlled by *qDTF-J* and at least two minor QTLs (Fig. 2 and Supplementary Fig. S1). Therefore, elevated *FT5a* expression may be one of the genetic factors that promote flowering under FR-enriched LD conditions. The NIL for the *ef* allele at *qDTF-J* developed from the cross between H-*e3* and J02 had higher *FT5a* expression than that for the *lf* allele, although both NILs had similar *E1* transcript abundances (Supplementary Fig. S3). Therefore, the level of *FT5a* expression was not directly correlated with that of *E1* expression, consistent with the finding that the DNA polymorphisms that increase *FT5a* expression may not affect the *cis* elements involved in the PHYA–E1 pathway.

Genetic factors activating *FT2a* and *FT5a* expression under inductive conditions for flowering remain poorly understood. The soybean *CO* orthologs *GmCOL1a*/*GmCOL1b* and *GmCOL2a*/*GmCOL2b* can fully complement the late-flowering phenotype of the Arabidopsis *co-1* mutant, suggesting that they are potential inducers of flowering in soybean (Wu *et al.*, 2014). However, GmCOL1a and GmCOL1b inhibit flowering under LD conditions (Cao *et al.*, 2015). This is similar to the rice (*Oryza sativa*) Heading date 1 (Hd1) protein, an ortholog of CO, which activates the expression of *Hd3a*, an ortholog of *FT*, under SD conditions, but suppresses *Hd3a* expression under LD conditions (Doi *et al.*, 2004). Further studies are needed to determine the transcription factor(s) that activate(s) the expression of both *FT2a* and *FT5a* under inductive conditions of flowering to improve our understanding of the molecular mechanisms of flowering in soybean. DNA polymorphisms between the *ef* and *lf* alleles detected in this study would be useful for identifying such transcriptional activator(s).

## Supplementary Data

Supplementary data are available at *JXB* online.

Table S1. Primers for DNA markers and sequencing.

Table S2. Primers for quantitative RT-PCR.

Table S3. Presence or absence of three indels in the promoter and 3′ UTR of *FT5a* in early-maturing soybean accessions.

Table S4. Variation of flowering time in accessions with the *ef* or *lf* alleles in different multi-locus genotypes at *E1*, *E2*, *E3* and *E4*.

Fig. S1. Two minor QTLs for reduced photoperiod sensitivity of Jiagedaqi-02.

Fig. S2. Relative levels of Glyma.16G043400 to Glyma.16G044000 mRNA at 35 DAE in near-isogenic lines for the *ef* and *lf* alleles at *qDTF-J*.

Fig. S3. Relative levels of *FT2a* and *E1* mRNA in near isogenic lines for the *ef* and *lf* alleles at *qDTF-J*.

Supplementary Data
