# Supplementary material for: A soybean quantitative trait locus that promotes flowering under long days is identified as FT5a, a FLOWERING LOCUS T ortholog
Source: J Exp Bot. 2016 Jul 15;67(17):5247–58. doi: 10.1093/jxb/erw283 (PMC5014162; doi:10.1093/jxb/erw283)
Supplement: Supplementary Data [file supp_67_17_5247__index.html]

A soybean quantitative trait locus that promotes flowering under long days is identified as FT5a, a FLOWERING LOCUS T ortholog — A soybean quantitative trait locus that promotes flowering under long days is identified as FT5a, a FLOWERING LOCUS T ortholog — Supplementary Data 

# A soybean quantitative trait locus that promotes flowering under long days is identified as *FT5a*, a *FLOWERING LOCUS T* ortholog

## Supplementary Data

Data files

- supplementary\_tables\_S1\_S4\_figures\_S1\_S3.pdf - Supplementary Data
