# Supplementary material for: A soybean quantitative trait locus that promotes flowering under long days is identified as FT5a, a FLOWERING LOCUS T ortholog
Source: J Exp Bot. 2016 Jul 15;67(17):5247–58. doi: 10.1093/jxb/erw283 (PMC5014162; doi:10.1093/jxb/erw283)
Supplement: Supplementary Data [file supp_erw283_supplementary_tables_S1_S4_figures_S1_S3.pdf]

Table S1. Primers for DNA markers and sequencing

Marker	Primer sequence	
	Forward (5′-3′)	Reverse (5′-3′)
DNA marker analysis		
SSR-J1	TCTCCCCCAGAGACATCAAT	GCAGCATGTGGTTAGTTAGT
SSR-J2	CTCAACATTAATGTCTTGGTG	TGTAATATCATGAGAGGGAT
SSR-J3	CCGTGTCCAATACATAGGTACA	GGGCCATTCATTCTGCTTGTGCT
SSR-J4	GCTGGGTGCTTCTGCGTAAC	CAGGACTTACACTCCTGTATG
SSR-J5	CAATATGACTGGAGGCTCATGA	CCTAAGTAGGCCTACCAAT
FT5a-Pro-indel	GTGTGGTCCATATTAATGCATAA	CGATCGGTCGATTCTTTTTGTT
FT5a-3′UTR-indel	CAAGTTCAATTAATGAAGATTC	TAAAACAAGGATAGCC
Sequencing analysis		
FT5a-CDS	TAGAACCCCAAACACAAACA	CAGCTGGAGTAAGGCATCCAA
FT5a-Pro1	GCAAGTTATAAGAGGGCATT	TTGGGCACTGATTGATTGTG
FT5a-Pro2	CACAATCAATCAGTGCCCAA	ATCGTGATCGGACGACAGAC
FT5a-Pro3	GTCTGTCGTCCGATCACGAT	TGTTTGTGTTTGGGGTTCTAC
FT5a-intron	TAGAACCCCAAACACAAACA	CCCTGTGCATCAATATTAAT
FT5a-3′UTR	ATTAATATTGATGCACAGGG	TCAAACGTACATACGTTTCTCTG

Table S2. Primers for quantitative RT-PCR

Target gene	Primer sequence		Reference
	Forward (5′-3′)	Reverse (5′-3′)	
E1	CACTCAAATTAAGCCCTTTCA	TTCATCTCCTCTTCATTTTTGTTG	Xia *et al*., 2012
FT2a	GGATTGCCAGTTGCTGCTGT	GAGTGTGGGAGATTGCCAAT	Kong *et al*., 2010
FT5a	TTGCAATGCTAGAGCATGCCATTC	CCCTGTAGTATGTATGAACCATGC	this study
TUB	GAGAAGAGTATCCGGATAGG	GAGCTTGAGTGTTCGGAAAC	Xu *et al*., 2013
Glyma.16G043300	GCCTTGATCCATGGCAAGAG	CACTGAAATATCCCAGCATAAGC	this study
Glyma.16G043400	CACTTAAAGAGAAGTGGGCTTCC	CCTCTAATTGAGACTTGAGAGTG	this study
Glyma.16G043500	GAGATTACGTTGGTGAGGCAAG	CCACACATACATATGGCTCCTCG	this study
Glyma.16G043600	CTCTCTGTAGGTGGGAAGTTCC	GCAATTCACACTTCAGTCTCTCG	this study
Glyma.16G043700	ACCATCACGCCGTTTTCGCG	TCTGACCGTTGGAGGCAGC	this study
Glyma.16G043800	AGGGATAGTAGATTTGGTTCTTCTG	CATTTTCACATCTGATTTTGCACGG	this study
Glyma.16G043900	TAGGATGAAGATGAGATGGAACTGA	GGAAAGAGGGGCAAAAGGG	this study
Glyma.16G044000	GAGTGACAGGTAGGGTGATCATT	GCTGCGATTTCAGTTCCTCCGT	this study

Table S3. Presence or absence of three indels in the promoter and 3′UTR of *FT5a* in early-maturing soybean accessions.

Accession	Origin	*qDTF-J* (*FT5a*) 41-bp segment in promoter	15-bp and 49-bp segments in 3′UTR	Allele	Day to flowering
Dongda 1	China	+	–	*ef*	38.0
Dongda 2	China	–	+	*lf*	42.0
Heihe 12	China	–	+	*lf*	42.5
Heihe 13	China	–	+	*lf*	42.5
Heihe 21	China	–	+	*lf*	40.3
Heihe 28	China	–	+	*lf*	42.7
Heihe 33	China	–	+	*lf*	42.5
Heihe 34	China	–	+	*lf*	48.5
Heihe 35	China	+	–	*ef*	39.0
Heihe 40	China	–	+	*lf*	42.3
Heihe 41	China	–	+	*lf*	39.0
Jiagedaqi 01	China	–	+	*lf*	40.8
Jiagedaqi 02	China	+	–	*ef*	45.0
Jiagedaqi 03	China	–	+	*lf*	42.5
Jiagedaqi 04	China	–	+	*lf*	41.3
Jiagedaqi 05	China	–	+	*lf*	41.0
Jiagedaqi 08	China	–	+	*lf*	41.3
Jiagedaqi 09	China	–	+	*lf*	41.5
Jiagedaqi 10	China	–	+	*lf*	43.3
Jiagedaqi 11	China	+	–	*ef*	42.5
Jiagedaqi 12	China	+	–	*ef*	41.3
Jiagedaqi 13	China	–	+	*lf*	42.5
Jiagedaqi 14	China	+	–	*ef*	42.0
Jiagedaqi 16	China	–	+	*lf*	41.0
Jiagedaqi 17	China	–	+	*lf*	40.7
Jiagedaqi 18	China	+	–	*ef*	40.0
Jiagedaqi 19	China	–	+	*lf*	40.7
Jiagedaqi 20	China	–	+	*lf*	53.5
Kamishunbetsu zairai	Japan	–	+	*lf*	37.0
Kamaishi 17	Japan	–	+	*lf*	39.0

Karafuto 1	Japan	—	+	*lf*	38.3
Ohfunato 45	Japan	—	+	*lf*	41.8
Ohyachi 2	Japan	—	+	*lf*	41.8
Okuhara 1	Japan	—	+	*lf*	38.8
Otome wase	Japan	+	—	*ef*	41.3
Sakamoto wase	Japan	+	—	*ef*	37.5
Darta	Poland	—	+	*lf*	40.5
Gai	Poland	—	+	*lf*	42.8
Nawiko	Poland	—	+	*lf*	40.0
Oktyabr-70	Russia	—	+	*lf*	43.0
Sadovy	Russia	—	+	*lf*	42.0
Sonata	Russia	—	+	*lf*	44.5
Vega	Russia	—	+	*lf*	41.5
Yubileinaya	Russia	—	+	*lf*	51.8
Zeika	Russia	—	+	*lf*	49.8
Zeya 2	Russia	+	—	*ef*	43.0
Kiev 242 BH	Ukraine	—	+	*lf*	42.8
Kiev 242 WH	Ukraine	—	+	*lf*	42.0
Ustya	Ukraine	—	+	*lf*	44.3
Yug 30	Ukraine	—	+	*lf*	40.8

+; presence, —; absence

The flowering time was evaluated in the natural daylength condition at Hokkaido University, Sapporo, Japan (43°06′N, 141°35′E) (Xu *et al.*, 2013).

Table S4. Variation of flowering time in accessions with the *ef* or *lf* alleles in different multi-locus genotypes at *E1*, *E2*, *E3*, and *E4*.

Multi-locus genotype[1)]	*qDTF-J*	Day to flowering									Total no. of accessions
		38	40	42	44	46	48	50	52	54	
e1/e2/e3/e4	*ef*										0
	lf	2			1						3
e1/e2/E3/e4	*ef*										0
	lf				2						2
e1/e2/e3/E4	*ef*	1									1
	lf			3	4						7
e1-as/e2/e3/e4	*ef*	1	2	2	2						7
	lf		3	12	4						19
e1-as/e2/e3/E4	*ef*					1					1
	lf					1		2	1	1	5
E1/e2/e3/e4	*ef*			1							1
	lf		2	2							4

1) The 50 accessions in Table S3 were classified into four multi-locus genotypes at *E1*, *E2*, *E3* and *E4*; the *e1*, *e3* and *e4* alleles contained different types of dysfunctional alleles (Xu *et al.*, 2013).

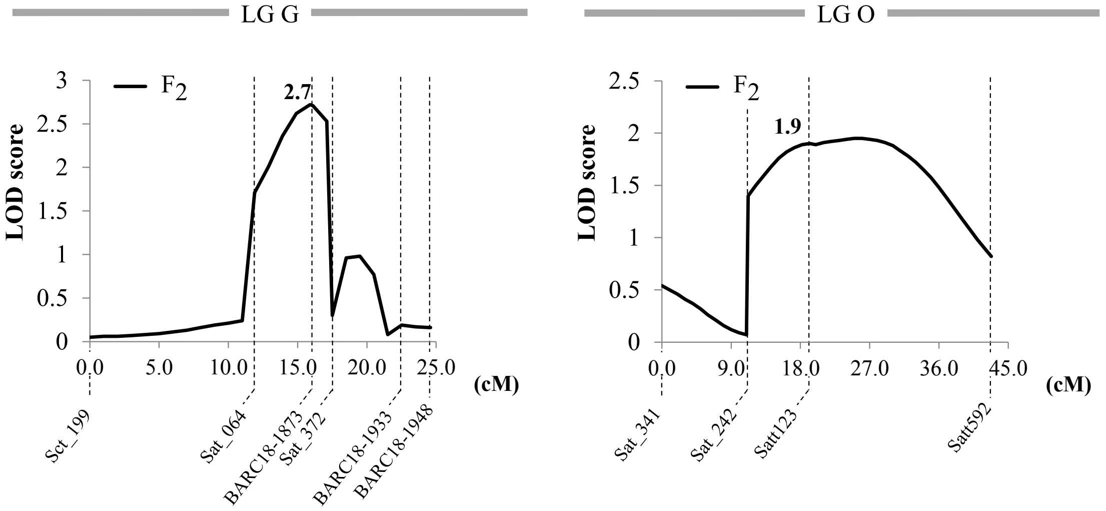

Fig. S1. Two minor QTLs for reduced photoperiod sensitivity of Jiagedaqi-02

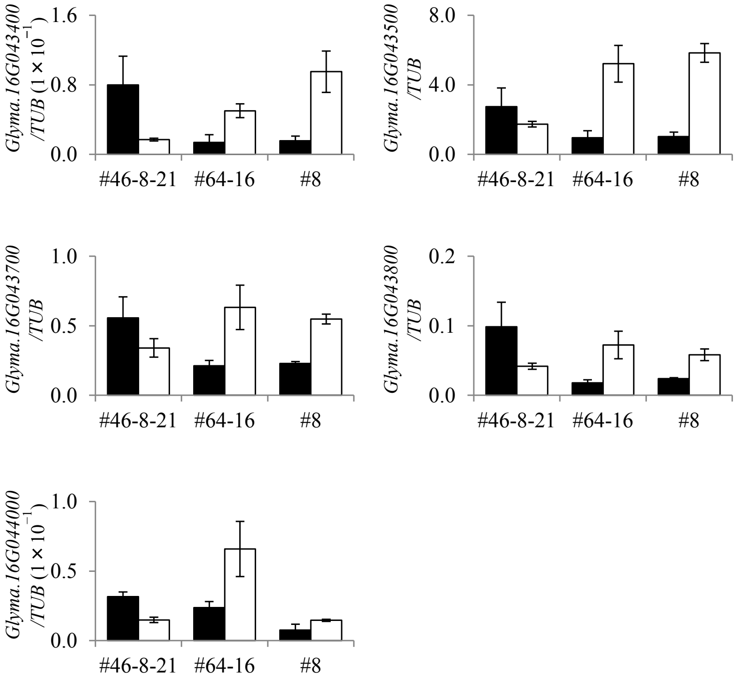

Fig. S2. Relative levels of Glyma.16G043400 to Glyma.16G044000 mRNA at 35 DAE in near-isogenic lines for the *ef* (closed bars) and *lf* (open bars) alleles at *qDTF-J*. Values are given relative to β-*tubulin* transcript levels. Error bars show standard error of the mean of three biological replicates (three independent plants). The transcript was not detected in Glyma.16G043300.

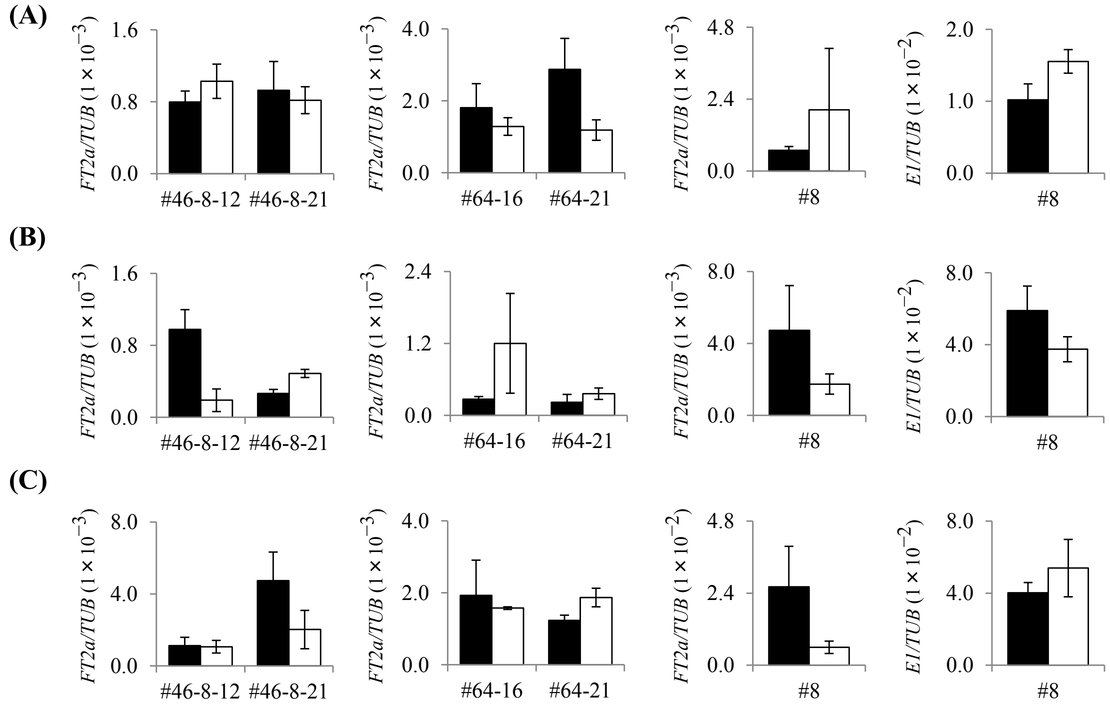

Fig. S3. Relative levels of *FT2a* and *E1* mRNA in near-isogenic lines for the *ef* (closed bars) and *lf* (open bars) alleles at *qDTF-J*. (A) – (C) Relative levels of *FT2a* and *E1* mRNA at (A) 15 DAE, (B) 25 DAE, and (C) 35 DAE. Four sets of near-isogenic lines (#46-8-12, #46-8-21, #64-16, and #64-21) developed from cross A exhibited low *FT2a* expression because they had the recessive *e9* allele with a *Ty1/copia*-like retrotransposon, *SORE-1*, inserted in the first intron of *FT2a* (Zhao *et al*., 2016). *FT2a* expression was also low in the near-isogenic line (#8) developed from cross B, likely because of the expression of *E1*. Values are given relative to β-*tubulin* transcript levels. Error bars show standard error of the mean of three biological replicates (three independent plants).
